# Safety and immunogenicity of the ChAdOx1 nCoV-19 (AZD1222) vaccine against SARS-CoV-2 in HIV infection: a single-arm substudy of a phase 2/3 clinical trial

**DOI:** 10.1016/S2352-3018(21)00103-X

**Published:** 2021-06-18

**Authors:** John Frater, Katie J Ewer, Ane Ogbe, Mathew Pace, Sandra Adele, Emily Adland, Jasmini Alagaratnam, Parvinder K Aley, Mohammad Ali, M Azim Ansari, Anna Bara, Mustapha Bittaye, Samantha Broadhead, Anthony Brown, Helen Brown, Federica Cappuccini, Enya Cooney, Wanwisa Dejnirattisai, Christina Dold, Cassandra Fairhead, Henry Fok, Pedro M Folegatti, Jamie Fowler, Charlotte Gibbs, Anna L Goodman, Daniel Jenkin, Mathew Jones, Rebecca Makinson, Natalie G Marchevsky, Yama F Mujadidi, Hanna Nguyen, Lucia Parolini, Claire Petersen, Emma Plested, Katrina M Pollock, Maheshi N Ramasamy, Sarah Rhead, Hannah Robinson, Nicola Robinson, Patpong Rongkard, Fiona Ryan, Sonia Serrano, Timothy Tipoe, Merryn Voysey, Anele Waters, Panagiota Zacharopoulou, Eleanor Barnes, Susanna Dunachie, Philip Goulder, Paul Klenerman, Gavin R Screaton, Alan Winston, Adrian V S Hill, Sarah C Gilbert, Andrew J Pollard, Sarah Fidler, Julie Fox, Teresa Lambe, Oxford COVID Vaccine Trial Group, Marion E.E. Watson, Rinn Song, Paola Cicconi, Angela M. Minassian, Sagida Bibi, Simon Kerridge, Nisha Singh, Catherine M. Green, Alexander D. Douglas, Alison M. Lawrie, Elizabeth A. Clutterbuck

**Affiliations:** a Oxford University Hospitals NHS Foundation Trust, Oxford, UK; b Peter Medawar Building for Pathogen Research, University of Oxford, Oxford, UK; c The Jenner Institute, University of Oxford, Oxford, UK; d Wellcome Centre for Human Genetics, University of Oxford, Oxford, UK; e Centre for Tropical Medicine and Global Health, University of Oxford, Oxford, UK; f Nuffield Department of Clinical Medicine and Oxford Vaccine Group, University of Oxford, Oxford, UK; g Department of Paediatrics, University of Oxford, Oxford, UK; h NIHR Oxford Biomedical Research Centre, Oxford, UK; i Department of Infectious Disease, Faculty of Medicine, Imperial College London, London, UK; j Department of HIV Medicine, St Mary's Hospital, Imperial College Healthcare NHS Trust, London, UK; k NIHR Imperial Clinical Research Facility and NIHR Imperial Biomedical Research Centre, London, UK; l Department of Infection, Harrison Wing and NIHR Clinical Research Facility, Guys and St Thomas' NHS Trust, London, UK; m NIHR Guy's and St Thomas' Biomedical Research Centre, London, UK; n Mahidol-Oxford Tropical Medicine Research Unit, Mahidol University, Bangkok, Thailand

## Abstract

**Background:**

Data on vaccine immunogenicity against SARS-CoV-2 are needed for the 40 million people globally living with HIV who might have less functional immunity and more associated comorbidities than the general population. We aimed to explore safety and immunogenicity of the ChAdOx1 nCoV-19 (AZD1222) vaccine in people with HIV.

**Methods:**

In this single-arm open-label vaccination substudy within the protocol of the larger phase 2/3 trial COV002, adults aged 18–55 years with HIV were enrolled at two HIV clinics in London, UK. Eligible participants were required to be on antiretroviral therapy (ART), with undetectable plasma HIV viral loads (<50 copies per mL), and CD4 counts of more than 350 cells per μL. A prime-boost regimen of ChAdOx1 nCoV-19, with two doses was given 4–6 weeks apart. The primary outcomes for this substudy were safety and reactogenicity of the vaccine, as determined by serious adverse events and solicited local and systemic reactions. Humoral responses were measured by anti-spike IgG ELISA and antibody-mediated live virus neutralisation. Cell-mediated immune responses were measured by ex-vivo IFN-γ enzyme-linked immunospot assay (ELISpot) and T-cell proliferation. All outcomes were compared with an HIV-uninfected group from the main COV002 study within the same age group and dosing strategy and are reported until day 56 after prime vaccination. Outcomes were analysed in all participants who received both doses and with available samples. The COV002 study is registered with ClinicalTrials.gov, NCT04400838, and is ongoing.

**Findings:**

Between Nov 5 and Nov 24, 2020, 54 participants with HIV (all male, median age 42·5 years [IQR 37·2–49·8]) were enrolled and received two doses of ChAdOx1 nCoV-19. Median CD4 count at enrolment was 694·0 cells per μL (IQR 573·5–859·5). No serious adverse events occurred. Local and systemic reactions occurring during the first 7 days after prime vaccination included pain at the injection site (26 [49%] of 53 participants with available data), fatigue (25 [47%]), headache (25 [47%]), malaise (18 [34%]), chills (12 [23%]), muscle ache (19 [36%]), joint pain (five [9%]), and nausea (four [8%]), the frequencies of which were similar to the HIV-negative participants. Anti-spike IgG responses by ELISA peaked at day 42 (median 1440 ELISA units [EUs; IQR 704–2728]; n=50) and were sustained until day 56 (median 941 EUs [531–1445]; n=49). We found no correlation between the magnitude of the anti-spike IgG response at day 56 and CD4 cell count (p=0·93) or age (p=0·48). ELISpot and T-cell proliferative responses peaked at day 14 and 28 after prime dose and were sustained to day 56. Compared with participants without HIV, we found no difference in magnitude or persistence of SARS-CoV-2 spike-specific humoral or cellular responses (p>0·05 for all analyses).

**Interpretation:**

In this study of people with HIV, ChAdOx1 nCoV-19 was safe and immunogenic, supporting vaccination for those well controlled on ART.

**Funding:**

UK Research and Innovation, National Institutes for Health Research (NIHR), Coalition for Epidemic Preparedness Innovations, NIHR Oxford Biomedical Research Centre, Thames Valley and South Midland's NIHR Clinical Research Network, and AstraZeneca.

## Introduction

SARS-CoV-2 emerged as a zoonotic virus late in 2019, is the causative agent of COVID-19, and, as of June 17, 2021, has been responsible for over 176 million confirmed cases and over 3 million deaths. The COVID-19 pandemic is likely to be brought under control only by a combination of public health interventions and effective vaccination. There are increasing data on the efficacy of different vaccines; however, most evidence for protection derives from studies of adults who are not immunocompromised.^1^, ^2^, ^3^ Although some countries are already reporting a beneficial effect of vaccination on COVID-19-associated morbidity and mortality,^4^ many are concerned that individuals with less functional immune systems (eg, those with cancer or recovering from organ transplantation) might have more severe disease^5^ and respond less well to vaccination.^6^

**Research in context**

**Evidence before this study**

Vaccination against SARS-CoV-2 reduces morbidity and mortality associated with COVID-19. The ChAdOx1 nCoV-19 (AZD1222) vaccine is safe, immunogenic, and efficacious; however, most data relating to vaccine outcomes have been derived from populations who are not immunocompromised, and responses for those with impaired immunity might not be as high and less well sustained. HIV infection might affect both the magnitude and durability of outcomes after vaccination and some people with HIV might require additional vaccine doses or adjusted regimens. We searched PubMed for studies published in English from database inception up to May 13, 2021, that assessed outcomes for people with HIV after vaccination against COVID-19 using the search terms “HIV” AND “COVID-19” OR “SARS-CoV-2” AND “vaccine” OR “vaccination”. We found no studies specifically reporting vaccine responses against SARS-CoV-2 for people with HIV.

**Added value of this study**

Our data are the first to show humoral and cell-mediated immune responses to the ChAdOx1 nCoV-19 (AZD1222) vaccine in people with HIV in a well characterised UK cohort with well suppressed viraemia and good CD4 cell counts. These data provide evidence that the vaccine is immunogenic in people with HIV on ART during early follow-up, but further data are needed to test durability of this response and in those who are viraemic or have low CD4 cell counts.

**Implications of all the available evidence**

These data add to the evidence that ChAdOx1 nCoV-19 (AZD1222) is likely to be protective and efficacious against COVID-19 for people with HIV, and reinforce the message that this population should be supported to receive vaccination against SARS-CoV-2.

For people living with HIV, successful antiretroviral therapy (ART) results in undetectable plasma HIV viraemia and restoration of CD4 cell numbers, improving mortality and morbidity.^7^ Clinically significant immune dysfunction can be reversed and prevented with daily ART,^8^ but full immune reconstitution might not be possible and many patients have persistent T-cell activation and exhaustion for many years.^9^, ^10^ Responses to vaccination for people with HIV might also be suboptimal despite ART, short-lived,^11^, ^12^ and require adjusted vaccine schedules.^13^ Because of the benefits of ART, non-replicating vaccines (including those licensed for SARS-CoV-2) can be given to all people with HIV, although replicating vaccines, such as yellow fever, should be avoided in those with CD4 counts below 200 cells per μL.^14^ An additional theoretical concern is that vaccine-induced immune activation can promote HIV viral transcription from latency with potential to increase the number of cells latently infected with HIV (the so-called viral reservoir),^15^ although the benefits of vaccination seem to outweigh this risk.

Early in the COVID-19 pandemic, no evidence existed that people living with HIV were at greater risk of infection or severe disease than those without HIV. More recently, several studies from countries, including the UK, North America, and South Africa,^16^, ^17^, ^18^, ^19^ have suggested otherwise. For people with HIV, and particularly those from Black and minority ethnic groups or with comorbidities, mortality due to COVID-19 has been speculated to be higher than for the general population.^16^ No studies have proven this hypothesis definitively, and the role of comorbidities (which might themselves be a result of HIV infection) remains a confounder. As a result, some groups have advocated to prioritisation of people with HIV for SARS-CoV-2 vaccination.^20^

Few data exist on immune responses to vaccination against SARS-CoV-2 in people with HIV. We studied the safety and immunogenicity of vaccination in participants with HIV on ART with CD4 counts of more than 350 cells per μL receiving two doses, 4–6 weeks apart, of a non-replicating chimpanzee adenovirus-vectored vaccine expressing the SARS-CoV-2 spike protein (ChAdOx1 nCoV-19), which has proven efficacy in people without HIV infection.^1^

## Methods

### Study design and participants

In this substudy, we studied a cohort of people living with HIV as an open-label non-randomised group within the larger multicentre phase 2/3 COV002 trial. This single-arm group comprised individuals with HIV who were stable on ART under routine follow-up at two London UK National Health Service (NHS) clinics and received ChAdOx1 nCoV-19 vaccination according to the schedule of attendance. Recruitment was done in HIV clinics at two centres in the UK (Imperial College NHS Trust and Guy's and St Thomas' NHS Foundation Trust).

Inclusion criteria for this substudy were age 18–55 years, a positive diagnosis of HIV infection, virological suppression on ART at enrolment (plasma HIV viral load <50 copies per mL), and a CD4 count of more than 350 cells per μL. The inclusion criteria for the COV002 trial have been published in full elsewhere.^1^

Written informed consent was obtained from all participants, and the trial was done in accordance with the principles of the Declaration of Helsinki and Good Clinical Practice. This study was approved in the UK by the Medicines and Healthcare products Regulatory Agency (reference 21584/0424/001-0001) and the South Central Berkshire Research Ethics Committee (reference 20/SC/0145). Vaccine use was authorised by Genetically Modified Organisms Safety Committees at each participating site.

### Clinical procedures

The ChAdOx1 nCoV-19 vaccine was produced as previously described^21^ and participants received two standard intramuscular doses given 4–6 weeks apart. Comparison was made with participants who were HIV negative, aged 18–55 years, enrolled into the main COV002 phase 2/3 randomised clinical trial, and randomly assigned (5:1) to receive either ChAdOx1 nCoV-19 or MenACWY by intramuscular vaccination. The dose of vaccine administered was the same across both groups. Only participants receiving the ChAdOx1 nCoV-19 vaccine were used for comparison. Full details of the COV002 HIV-negative cohort have been published previously.^22^

Before enrolment, all participants attended a screening visit where a full medical history and examination were done, in addition to blood tests to exclude biochemical or haematological abnormalities (full blood count; kidney and liver function tests). Participants with a history of laboratory-confirmed SARS-CoV-2 infection by anti-N protein IgG immunoassay (Abbott Architect, Abbott Park, IL, USA) at screening were excluded. Subsequent study visits were scheduled at days 0 (vaccine prime), 7, 14, 28 (vaccine boost), 31, 35, 42, and 56. Participants were asked to complete diaries reporting solicited systemic and local adverse reactions for 7 days after both their prime and boost vaccinations. Adverse events were recorded at all visits. Blood samples for immunological analysis were collected on days 0, 14, 28, 42, and 56.

### Laboratory procedures

Peripheral blood mononuclear cells (PBMCs) were isolated by density gradient centrifugation using Lymphoprep (Stem Cell Technologies, Cambridge, UK). PBMCs were collected and washed twice with pre-warmed R10 medium: Roswell Park Memorial Institute (also known as RPMI) 1640 medium (Sigma Aldrich, St Louis, MO, USA) supplemented with 10% heat-inactivated fetal calf serum (FCS; Sigma), 1 mM penicillin-streptomycin solution (Sigma), and 2 mM L-glutamine solution (Sigma). After the second centrifugation, cells were resuspended in R10 and counted using the Guava ViaCoun assay (Guava Technologies Hayward, CA, USA) on the Muse Cell Analyzer (Luminex Cooperation). T-cell enzyme-linked immunospot assay (ELISpot) assays were done on freshly isolated PBMCs, and CellTrace Violet (CTV; Life Technologies) cell proliferation assays (CTV; ThermoFisher Scientific, CA, USA) were done on frozen samples.

Humoral responses at baseline and after vaccination were assessed using a standardised total IgG ELISA against trimeric SARS-CoV-2 spike protein as described previously.^21^ Briefly, Nunc MaxiSorp 96-well ELISA plates (Life Technologies, Paisley, UK) were coated with 2 μg/mL of full-length trimerised SARS-CoV-2 spike glycoprotein and stored at 4°C overnight for at least 16 h. After coating, plates were washed six times with phosphate-buffered saline (PBS) with 0·05% Tween (Merck Life Science UK, Dorset, UK) and blocked with casein for 1 h at room temperature. Thawed samples were treated with 10% Triton X-100 (Merck Life Science UK, Dorset, UK) for 1 h at room temperature and subsequently diluted in casein and plated in triplicate for incubation for 2 h at room temperature alongside two internal positive controls (controls 1 and 2) to measure plate-to-plate variation. Control 1 was a dilution of convalescent plasma sample and control 2 was a research reagent for anti-SARS-CoV-2 antibody (code 20/130 supplied by National Institute for Biological Standards and Control, Herts, UK). The standard pool was used in a two-fold serial dilution to produce ten standard points that were assigned arbitrary ELISA units (EUs). Goat anti-human IgG (γ-chain specific) conjugated to alkaline phosphatase was used as secondary antibody and plates were developed by adding 4-nitrophenyl phosphate in diethanolamine substrate buffer (Fisher Scientific UK, Loughborough, UK). An ELx808 microplate reader (BioTek Instruments; Winooski, VT, USA) was used to provide optical density measurement of the plates at 405 mm. Standardised EUs were determined from a single dilution of each sample against the standard curve, which was plotted using the 4-Parameter logistic model (Gen5 version 3.09; BioTek Instruments, Winooski, VT, USA). Each assay plate consisted of samples and controls plated in triplicate, with ten standard points in duplicate and four blank wells.

Antibody neutralisation was measured in a randomly selected subset of participants by use of a focus reduction neutralisation test (FRNT), as described previously,^23^ where the reduction in the number of the infected foci is compared with a no antibody negative control well. Briefly, serially diluted antibody or plasma was mixed with the SARS-CoV-2/human/AUS/VIC01/2020 strain and incubated for 1 h at 37°C. The mixtures were then transferred to a 96-well, cell culture-treated, flat-bottom microplate containing confluent Vero cell monolayers in duplicate and incubated for further 2 h at 37°C, followed by the addition of 1·5% semi-solid carboxymethyl cellulose overlay medium to each well to limit virus diffusion. A focus forming assay was then done by staining Vero cells with human anti-nucleoprotein monoclonal antibodies (mAb206) followed by peroxidase-conjugated goat anti-human IgG (A0170; Sigma). Finally, the foci (infected cells), approximately 100 per well in the absence of antibodies, were visualised by adding TrueBlue peroxidase substrate (SeraCare, Milford, MA, USA; #5510-0030). Virus-infected cell foci were counted on an Autoimmun Diagnostika (AID) ELISpot Reader System using AID ELISpot software 7.0 (AID Autoimmun Diagnostika; Straßberg, Germany). The proportion of focus reduction was calculated and half maximal inhibitory concentration (IC50; reported as FRNT50) was determined using the probit program from the IBM SPSS Statistics 27 package.

ELISpot assays were done as described previously^21^ using a validated protocol with freshly isolated PBMCs to determine responses to the SARS-CoV-2 spike vaccine antigen at days 0 (before vaccination), 14, 28 (on the day of boost), 42, and 56. Assays were done using Multiscreen IP ELISpot plates (Merck Millipore, Watford, UK) coated with 10 μg/mL of human anti-IFN-γ antibody and developed using streptavidin alkaline phosphatase antibody conjugate kits (Mabtech, Stockholm, Sweden) and BCIP NBT-plus chromogenic substrate (Moss, Pasadena, MA, USA). PBMCs were separated from whole blood with lithium heparin by density centrifugation within 4 h of venepuncture. Cells were incubated for 18–20 h in RPMI (Sigma) containing 1000 units per mL penicillin, 1 mg/mL streptomycin solution, and 10% heat-inactivated, sterile-filtered FCS, previously screened for low reactivity (Labtech International, Healthfield, East Sussex, UK) with a final concentration of 10 μg/mL of each peptide. A total of 253 synthetic peptides (15mers overlapping by ten amino acids) spanning the entire vaccine insert, including the tissue plasminogen activator (tPA) leader sequence, were used to stimulate PBMCs (ProImmune, Oxford UK). Peptides were pooled into 12 pools for the SARS-CoV-2 spike protein containing 18–24 peptides, plus a single pool of five peptides for the tPA leader. Peptides were tested in triplicate, with 2·5 × 10^5^ PBMCs added to each well of the ELISpot plate in a final volume of 100 μL. Results are expressed as spot forming cells (SFCs) per million PBMCs, calculated by subtracting the mean negative control response from the mean of each peptide pool response and then summing the response for the 12 peptide pools spanning S1 and S2. Staphylococcal enterotoxin B (0·02 μg/mL) and phytohaemagglutinin-L (10 μg/mL) were pooled and used as a positive control. Plates were counted using an AID automated ELISpot counter (Autoimmun Diagnostika GmbH, algorithm C) using identical settings for all plates, and counts were adjusted only to remove artifacts. A lower limit of detection of 48 SFCs per million PBMCs was determined on the basis of the minimum number of spots that could be detected. To define a positive response for categorical analyses, a definition of greater than 221 SFCs per million PBMCs was used.

Cryopreserved PBMCs were used for the T-cell proliferation assay, which measures the decrease in CTV dye in proliferating cells after antigen stimulation, as described previously.^24^ Briefly, PBMCs were thawed and washed twice with 1 mL of PBS followed by labelling with CTV at a final concentration of 2·5 μM for 10 min at room temperature. The labelling reaction was quenched with 4 mL of fetal bovine serum at 4°C and cells were resuspended in RPMI medium supplemented with 10% human blood group type AB serum (Sigma), 1 mM penicillin-streptomycin solution, and 2 mM L-glutamine solution, and subsequently plated in a 96-well round bottom plate at a plating density of 0·25 × 10^6^ cells per well in duplicate wells (total of 0·5 × 10^6^ cells per condition). Cells were stimulated with peptide pools spanning SARS-CoV-2 spike (S1 and S2) at a final concentration of 1 μg/mL per peptide. For antigenic control, class 1 and 2 optimal peptides for FEC-T (flu, EBV, CMV, and tetanus) were pooled at a final concentration of 1 μg/mL per peptide. Media, containing 0·1% dimethyl sulfoxide (DMSO; Sigma) representing DMSO content in peptide pools, was used as a negative control and 2 μg/mL phytohaemagglutinin L (Sigma) was used as positive control. Cells were then incubated at 37°C, with 5% carbon dioxide and 95% humidity for 7 days, with a change of media on day 4. At the end of the incubation period, cells were stained using anti-human CD3, CD4, CD8, and a live cell discriminator (Live/Dead near Infra-red, Life Technologies; ThermoFisher Scientific, CA, USA). All samples were acquired using a BD Fortessa X20 (BD Bioscience, San Jose, CA, USA) or MACSQuant x10 (Miltenyi Biotec, Bergisch Gladbach, Germany). Responses above 1% were considered true positive. All datapoints presented represent a single participant and are presented as background subtracted data. Gating strategy is shown in the appendix (p 5).

For ex-vivo T-cell immune activation assays, cryopreserved PBMCs were thawed in 30 mL of R10 media. Cells were counted and rested for 1 h at a cell density of 2 × 10^6^ per mL of R10 medium and 1 μL of benzonase nuclease (EMD Millipore; Sigma Aldrich) per mL of R10. Following rest, 2–3 million cells were used. Cells were washed in staining buffer (Biolegend, San Diego, CA, USA) and then blocked with FC receptors blocker (FcX blocker, Biolegend, San Diego, CA, USA) for 10 min at room temperature, followed by live-cell staining using L/D (Live/Dead) Aqua (ThermoFisher Scientific, CA, USA). All cells were then washed with cell staining buffer. Antibodies for assessing immune activation by flow cytometry were used as a cocktail and added to the cell pellet. Cells were subsequently incubated at 37°C for 15 min, followed by a wash and fixation in 4% paraformaldehyde (Sigma Aldrich) for 10 min at room temperature. Paraformaldehyde was washed off and cells were resuspended in PBS for acquisition. The gating strategy for T-cell activation is shown in the appendix (p 6).

### Outcomes

The primary outcomes for the cohort of people living with HIV were safety and reactogenicity of the vaccine as determined by serious adverse events and solicited local and systemic reactions recorded in electronic diaries. As per the protocol, serious adverse events occurring throughout the study period, solicited local and systemic reactions occurring within 7 days of each dose, and unsolicited adverse events occurring within 28 days of each dose were recorded. The secondary outcome of interest in this cohort was the immunogenicity profile of ChAdOx1 nCoV-19 in people living with HIV. Here, we present antibody responses (serology and neutralisation) and T-cell responses (ELISpot and CTV) as well as T-cell activation and exhaustion data up to 56 days after prime vaccination. Further analyses and timepoints will be published when available.

### Statistical analysis

This study was not powered to a specific endpoint and the sample size was based on practical recruitment considerations in line with other subgroups of the COV002 study. We analysed all outcomes in all participants who received both doses of the vaccination schedule and with available samples, unless otherwise specified. We describe safety endpoints as frequencies and proportions. We compared solicited adverse events using the χ^2^ test. We log-transformed serological, FRNT50, and ELISpot data for analysis. FRNT50 titres less than 20 were given the value 10 for statistical analysis. We present medians and IQRs for immunological endpoints. We used non-parametric analysis (Spearman) for correlations between two immunological endpoints. For log-transformed correlations, we used parametric analyses (Pearson). For comparison of two non-parametrically distributed unpaired variables, we used the Wilcoxon rank sum (Mann Whitney *U*) test, or Friedman test for repeated measures. For comparison of two non-parametrically distributed paired datasets, we used the Wilcoxon matched-pairs signed rank test. We used the χ^2^ test for comparison of ELISpot responses.

Missing data were not imputed. We did all analyses using R (version 3.6.1 or later), and Prism 9 (GraphPad Software). The COV002 study is registered with ClinicalTrials.gov, NCT04400838, and is ongoing.

### Role of the funding source

AstraZeneca reviewed the data from the study and the final manuscript before submission, but the authors retained editorial control. All other funders of the study had no role in study design, data collection, data analysis, data interpretation, or writing of the report.

## Results

Between Nov 5 and Nov 24, 2020, 54 individuals with HIV were enrolled in the study and received ChAdOx1 nCoV-19 prime and boost vaccinations between 4–6 weeks apart. All participants were male, with a median age of 42·5 years (IQR 37·2–49·8). Most participants self-reported White ethnicity, with others reporting as Asian, Mixed, or other (table). All participants were receiving suppressive ART (viral load <50 HIV RNA copies per mL of plasma) and the median CD4 count was 694·0 cells per μL (IQR 573·5–859·5; appendix p 8).

**Table:** Cohort demographics in participants who were given ChAdOx1 nCoV-19

		**HIV-positive cohort (n=54)**	**HIV-negative cohort (n=50)**
Sex			
	Male	54 (100%)	26 (52%)
	Female	0	24 (48%)
Age, years		42·5 (37·2–49·8)	38·5 (29·2–45·0)
Ethnicity			
	White	44 (81%)	40 (80%)
	Black	0	1 (2%)
	Asian	2 (4%)	8 (16%)
	Mixed	4 (7%)	0
	Other	4 (7%)	1 (2%)
	Missing	0	0
On antiretroviral therapy		54 (100%)	..
Plasma HIV viral load		<50·0*	..
CD4 count >350 cells per μL		694·0 (573·5–859·5)	..

Data are n (%) or median (IQR). Data are for participants with HIV and without HIV included in these analysis. Participants with HIV were recruited specifically for this study, whereas the HIV-negative group are historical controls.^25^

* All viral loads were lower than 50 RNA copies per mL of plasma.

**Figure 1:**
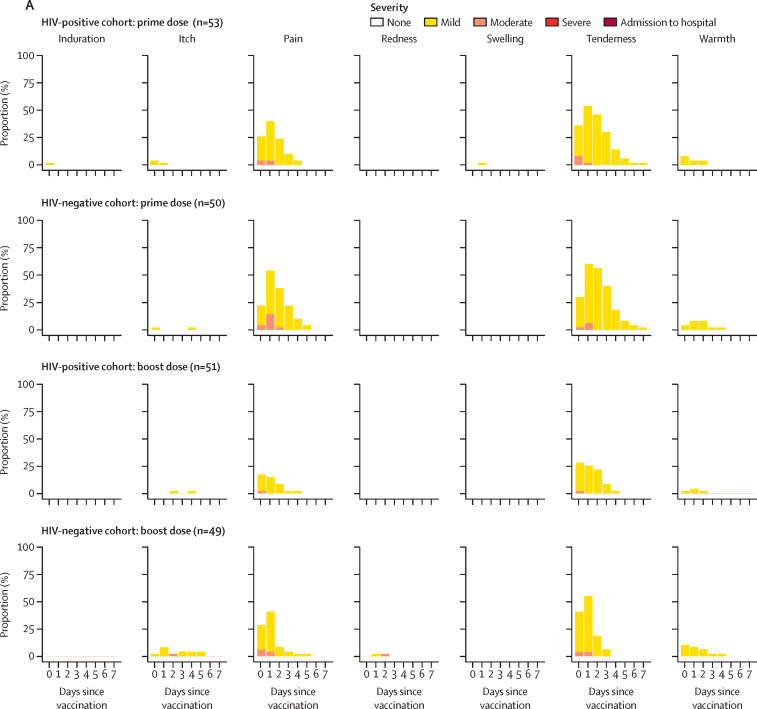

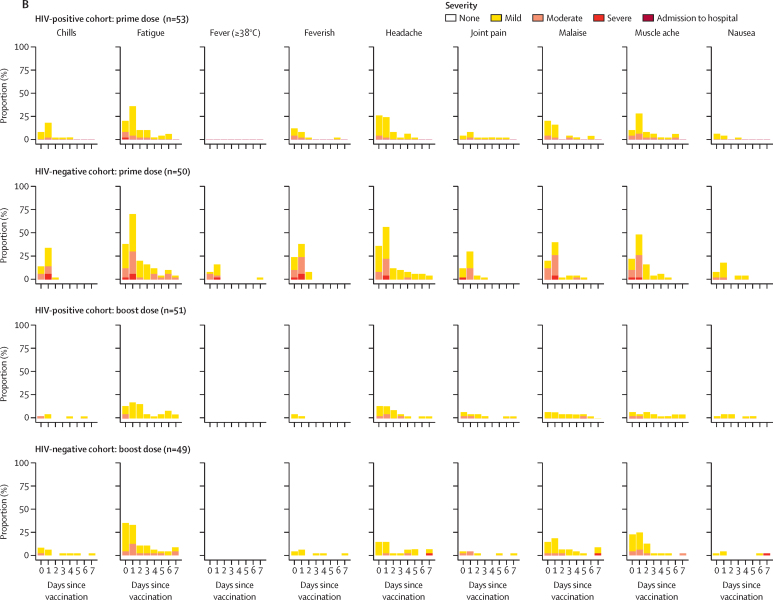
Solicited local (A) and systemic (B) adverse events in participants vaccinated with ChAdOx1 nCoV-19 Solicited adverse reactions in the first 7 days after vaccination, as recorded in participant symptom electronic diaries. Day 0 is the day of vaccination. Vertical bars show proportion of participants reporting symptoms. Bars are colour-coded to show levels of severity.

All 54 participants completed the vaccination schedule. No serious adverse events were reported. Solicited adverse events from the first 7 days after prime and boost vaccination were self-reported using participant diaries by 53 (98%) of 54 participants after prime dose, and 51 (94%) of 54 after boost dose. After a prime dose of ChAdOx1 nCoV-19, 26 (49%) participants with HIV reported pain at the site of vaccination that was mild or moderate in severity (figure 1A; appendix p 2). Headache and fatigue were the most reported systemic reactions, and were reported by more participants after the prime dose (both 25 [47%] of 53) than after the boost dose (12 [24%] of 51 with headache and 15 [29%] of 51 with fatigue; figure 1B; appendix p 2). Other reactions after the prime dose were chills in 12 (23%) participants, joint pain in five (9%) participants, malaise in 18 (34%) participants, muscle aches in 19 (36%) participants, feverish in ten (19%) participants, and nausea in four (8%) participants. A similar pattern of solicited adverse events was reported in participants with and without HIV, although a lower incidence of adverse events was observed after a boost dose in people living with HIV than in those without HIV (appendix p 3).

**Figure 2:**
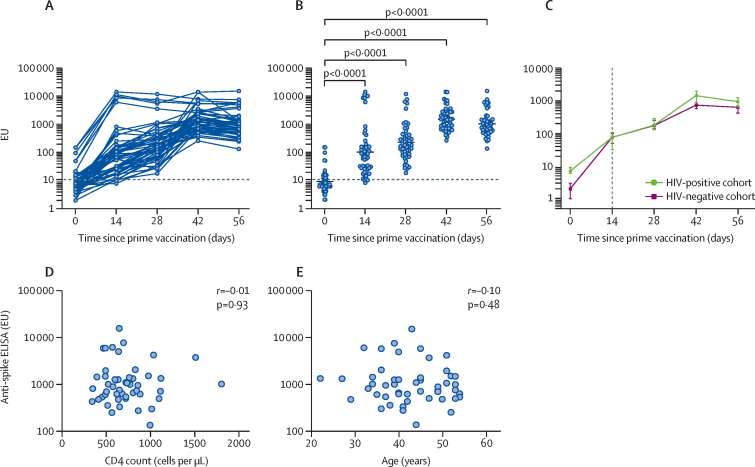
Serological responses to vaccination with ChAdOx1 nCoV-19 in people with HIV SARS-CoV-2 IgG response by standardised ELISA to spike protein in trial participants show individual (A) and grouped (B) responses at days 0, 14, 28, 42, and 56 after vaccination. The threshold for a positive response is shown by the hashed line at 10 EU; horizontal bars show median values. (C) Comparison between HIV-positive and HIV-negative cohorts. Datapoints are medians, with error bars showing 95% CIs. The vertical line at day 28 marks the timing of the booster dose. Plots of anti-spike ELISA on day 56 after vaccination *vs* CD4 cell count (D) and age (E). Statistics calculated using Spearman's correlation coefficient. Exact numbers of participants who provided data at each timepoint are provided in the appendix (p 3). EU=ELISA units.

Antibodies against the SARS-CoV-2 spike protein peaked at day 42 (median 1440 EUs [IQR 704–2728]; n=50) after the prime dose (14 days after the boost dose), and were sustained to day 56 (median 941 EUs [531–1445]; n=49; figure 2A, B; appendix p 3). In comparison with HIV-negative participants, we found no difference in responses at days 14 and 28, although responses in the HIV-positive cohort were significantly higher at days 42 and 56 (figure 2C; appendix p 3). At day 56, we saw no correlation with antibody response and CD4 cell count (Spearman's correlation *r*=–0·01; p=0·93; figure 2D) or age (Spearman's correlation *r*=–0·10; p=0·48; figure 2E).

**Figure 3:**
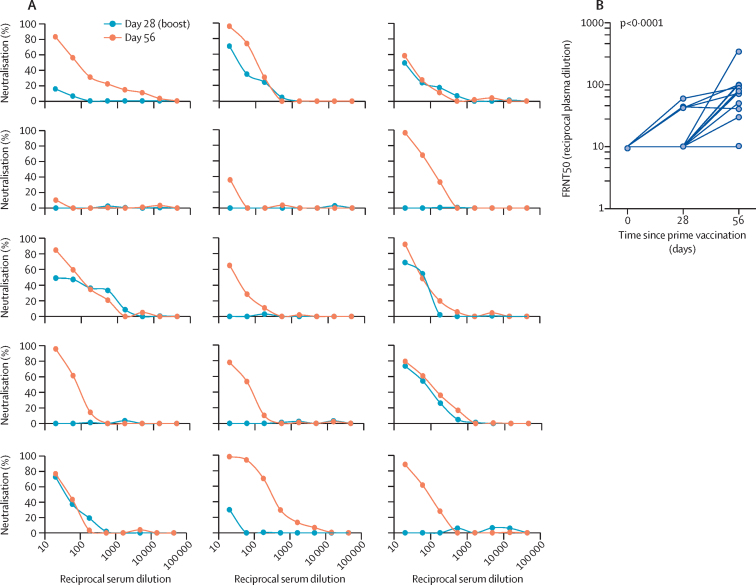
Neutralisation of SARS-CoV-2/human/AUS/VIC01/2020 strain (A) Neutralisation of SARS-CoV-2 measured using an FRNT using plasma for 15 randomly selected trial participants with HIV at day 28 boost (blue) and day 56 (orange). Datapoints are mean values at each reciprocal serum dilution. (B) FRNT50 values for the 15 participants at days 0, 28, and 56 after prime vaccination. Undetectable neutralisation is reported as less than 20 FRNT50, and the value 10 was allocated for presentation and analysis. p value calculated using Friedman test. FRNT=focus reduction neutralisation test.

In a randomly selected subset of 15 participants, neutralisation of the SARS-CoV-2/human/AUS/VIC01/2020 strain was measured by FRNT at day 28 (before boost dose) and day 56 after prime vaccination (figure 3A). By day 28, four (27%) of 15 participants showed evidence of neutralisation, increasing to 13 (87%) participants by day 56, with median FRNT50 values of 10·0 (IQR 10·0–42·0) at day 28 and 75·0 (30·0–100·0) at day 56 (p<0·0001; figure 3B). We observed positive correlation between serology and neutralisation at day 56 (Pearson's *r*=0·75; p=0·0013; appendix p 7).

**Figure 4:**
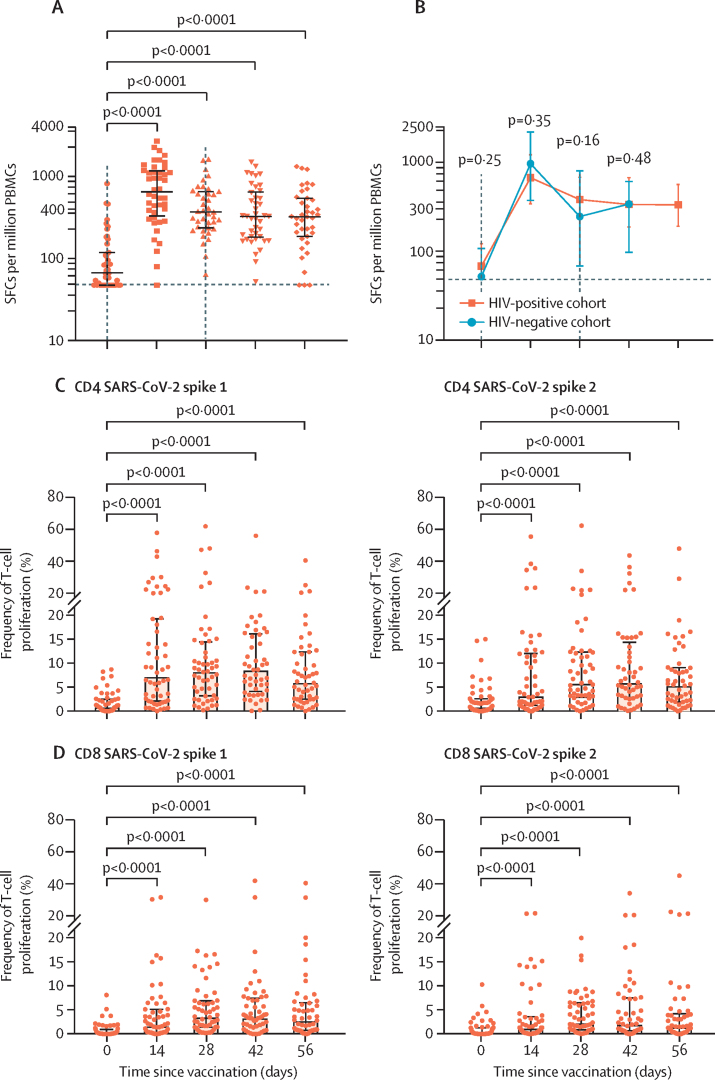
T-cell responses to vaccination with ChAdOx1 nCoV-19 in people living with HIV Time course of IFN-γ ELISpot responses to peptides spanning the SARS-CoV-2 spike vaccine insert for participants with HIV (A) and compared with HIV-negative cohort (B). In panel A, datapoints are readings per participant at each timepoint and the thick horizontal bar shows the median, with error bars showing the IQR; and in panel B, each datapoint is the median of each cohort at each timepoint, with error bars showing the IQR. In panels A and B, the lower limit of detection is indicated with the horizontal dotted line and is set at 48 SFCs per million PBMCs, and vertical dotted lines indicate vaccination timepoints. For panel A, statistical analysis was completed using Wilcoxon signed rank tests and in panel B statistical analysis was completed using Wilcoxon rank sum tests. Frequency of CD4 (C) and CD8 (D) proliferating T cells in response to stimulation by overlapping peptide pools spanning spike S1 and S2. In panels C and D, datapoints represent each participant, and the bars and error bars show the overall median and IQR, and p values were calculated using the Wilcoxon signed rank test. Exact numbers of participants who provided data at each timepoint are provided in the appendix (p 4). ELISpot=enzyme-linked immunospot assay. PBMCs=peripheral blood mononuclear cells. SFC=spot forming cells.

Summed IFN-γ ELISpot responses across 12 pools of SARS-CoV-2 spike peptides peaked on day 14 after prime dose (median 674 SFCs per million PBMCs [IQR 341–1223]; n=44) and were sustained at a lower level until day 56 (median 333 SFCs per million PBMCs [191–564]; n=39). At all timepoints, responses were significantly increased compared with baseline (figure 4A). No difference was found between the HIV-positive cohort and the HIV-negative cohort in COV002 (Wilcoxon rank sum test p>0·05 at all timepoints; figure 4B). 14 days after vaccination, 39 (89%) of 44 HIV-positive individuals with available samples and 25 (81%) of 31 HIV-negative individuals with available samples had a response (defined as >221 SFCs per million PBMCs) by ELISpot (Wilcoxon rank-sum/Mann Whitney test p=0·30). By day 42, 28 (67%) of 42 HIV-positive individuals and 17 (61%) of 28 HIV-negative individuals had a response (Wilcoxon rank-sum/Mann Whitney test p=0·74; appendix p 4). We then turned to the CTV T-cell proliferation assay as a potentially more sensitive measure of T-cell responses and with the ability to discriminate between CD4 and CD8 cells. Results are presented as responses to two peptide pools (S1 and S2), between them covering the SARS-CoV-2 spike. Consistent with the ELISpot data, we found significant increases in responses after vaccination for both CD4 and CD8 cells. Responses to the control FEC-T and phytohaemagglutinin-L pools were unchanged across the study (appendix p 7). Proliferative CD4 cell responses to SARS-CoV-2 spike peaked at day 42 for pool S1 (median 8·64% [IQR 4·00–16·21]) and day 28 for pool S2 (5·78 [2·71–12·18]; figure 4C). For CD8 cells, proliferative responses were less than for CD4 cells, and peaked on day 28 for both pools (median 3·55% [IQR 1·40–6·84] for S1 and 2·33% [0·85–6·33] for S2; figure 4D).

All participants were screened for evidence of previous SARS-CoV-2 infection using the anti-nucleoprotein immunoassay and excluded if positive. We identified six individuals who tested negative for SARS-CoV-2 at screening but had high baseline anti-spike serological responses (≥20 EU) and had a strong responses to vaccination (figure 2A, B). To determine whether these individuals might have undiagnosed previous infection, we explored the ELISpot data to see whether there was evidence of T-cell immunity pre-vaccine. However, these individuals did not have a noticeably increased baseline ELISpot responses compared with the rest of the HIV-positive cohort. Indeed, we found no correlation between baseline serological and ELISpot responses across the whole study group (Spearman's *r*=0·23; p=0·12; appendix p 8).

Chronic immune activation in HIV-positive individuals might affect vaccine responses. Additionally, vaccination might itself lead to T-cell activation and increased HIV viral transcription from the latent reservoir. To explore this, co-expression of CD38 and HLA DR was used as a marker of CD4 and CD8 cell activation and was measured at all timepoints. We found a small but significant increase in immune action for both CD4 and CD8 cells at day 14 after prime vaccination, but this response was not sustained beyond this timepoint (appendix p 9). Additionally, immune activation at baseline (on CD4 or CD8 cells) had no effect on subsequent serological or cell-mediated vaccine responses (appendix p 8).

## Discussion

These preliminary data show that the ChAdOx1 nCoV-19 vaccine given as prime-boost dosing given 4–6 weeks apart was well tolerated and produced equivalent immune responses in people living with HIV who are well controlled on ART compared with a similar adult population without HIV. These findings suggest that no dose adjustment in the vaccine is needed for people with HIV on ART with CD4 counts of more than 350 cells per μL. Although this study was too small to report protection from infection, the measured immunological responses are similar to those seen in larger studies of HIV-negative participants for whom there is increasing evidence that vaccination leads to a reduction in symptomatic cases and hospital admissions,^25^, ^26^, ^27^ including to variants of concern such as B.1.1.7.^28^

As of June 17, 2021, vaccine roll-out against SARS-CoV-2 is continuing in all countries, and although there are concerns over the potential impact of new variants, there are clear beneficial impacts on hospital admissions and deaths related to COVID-19.^5^, ^28^, ^29^, ^30^ However, individuals with primary or secondary immunodeficiencies are a population of particular concern because they have increased rates of COVID-19-related morbidity and mortality and might respond less well to vaccination. For people with HIV, the relative risk of disease progression, admission to hospital, or death related to COVID-19 is not clear, with several observational studies reporting different results.^16^, ^17^, ^18^, ^19^, ^29^, ^30^ This is likely partly related to the heterogeneity of outcomes for individuals with HIV, contingent on access to effective suppressive ART. We studied participants at two major UK NHS centres for HIV care, all of whom were on long-term ART with undetectable viraemia and CD4 counts of more than 350 cells per μL.

Vaccination with ChAdOx1 nCoV-19 had a favourable safety profile with no serious adverse events, and symptoms that were reported within 7 days of vaccination were generally mild or moderate. The pattern of reactogenicity was very similar to a population without HIV who received the same vaccine schedule. Although some solicited symptoms appeared to be less frequent for people living with HIV, the comparator group without HIV were an earlier cohort of participants who had been vaccinated in the context of a single-blind randomised study^25^ and were not advised to take paracetamol if symptomatic after vaccination. Additionally, the HIV-positive cohort were all male, compared with only 52% being male in the HIV-negative cohort, so the findings might have been affected by sex differences in reactogenicity profiles.

The participants in this study had both serological and cell-mediated immune responses to the SARS-CoV-2 spike protein after vaccination and we found no significant difference in responses in our HIV-positive cohort compared with HIV-negative individuals. However, data were available to compare only the anti-spike ELISA and T-cell ELISpot responses with this control group, and do not include details of T-cell polyfunctionality. Additional supportive evidence was provided by the neutralisation assays (done on a subset of participants by use of antibody-mediated live virus neutralisation^23^) and T-cell proliferation assays (done on all available samples), which together supported a rapid response to vaccination that was sustained up to 56 days.

Our study had several limitations. First, these are preliminary data reporting results up to 56 days after vaccination. Further follow-up will take place after 6 and 12 months to determine how the vaccine responses are sustained in people with HIV, which is of importance in light of evidence of shorter-lived responses to some vaccines in this population.^31^ However, in view of concerns regarding the potential for worse outcomes with COVID-19 for people with HIV combined with anxiety over less effective immune responses to vaccination, these preliminary data showing good early immunity are reassuring. Second, this was an open-label study without randomisation and there was no parallel recruitment of adults without HIV at the same centres. Instead, we used data from adults without HIV recruited to the COV002 trial using the same vaccine protocol. Although there was an imbalance in the sex distribution of the people with HIV study group, the cohorts were otherwise similarly matched for age and ethnicity. We have previously found equivalent responses in males and females using this vaccine protocol,^32^ and a South African study in which 69% of a cohort of individuals with HIV receiving vaccination were female showed similar results.^33^ Accordingly, we do not think the sex difference in this study had a substantial effect on the outcome.

Extrapolation of the data in our study to all people with HIV regardless of CD4 cell count, gender, ethnicity, and ART status should be done with caution. Our cohort comprised predominantly White European men with access to long-term ART and high CD4 cell counts. Encouragingly, interim data from South Africa also suggest favourable safety and immunogenicity data for vaccination with ChAdOx1 nCoV-19 for people with HIV.^33^ One might argue that in a population without ART who were viraemic or had CD4 counts below 350 cells per μL the results might have been different. More data are needed in these groups to support our findings in individuals who might be considered to have optimal HIV management, and to factor in data on comorbidities, duration of HIV infection, ART history, and CD4/CD8 ratios. Regardless, vaccination against SARS-CoV-2 should continue to be offered and encouraged for all those living with HIV.

In this study, we focused on responses to the ChAdOx1 nCoV-19 vaccine, so we cannot comment on potential responses among people living with HIV to other SARS-CoV-2 vaccines, or to other vaccine interval durations. Other studies have shown that vaccines that induce similar immunological responses to those we document here provide protection from both infection and disease progression.^1^, ^2^, ^3^ Further studies are needed in people with HIV and other groups whose immune responses to vaccination might be suboptimal. However, our findings of robust immune responses to the ChAdOx1 nCoV-19 vaccine, irrespective of HIV status, are encouraging, and reinforce the message that people living with HIV should be supported to receive vaccination.

## Data sharing

Anonymised participant-level data will be made available when the trials are complete, upon requests directed to the corresponding author. Proposals will be reviewed and approved by the sponsor (Oxford University), investigator, and collaborators on the basis of scientific merit. After approval of a proposal, data can be shared through a secure online platform after signing a data access agreement. All data will be made available for a minimum of 5 years from the end of the trial.

## Declaration of interests

Oxford University has entered into a partnership with AstraZeneca for further development of ChAdOx1 nCoV-19 (AZD1222). SCG is cofounder of Vaccitech (a collaborator in the early development of this vaccine candidate) and named as an inventor on a patent covering use of ChAdOx1-vectored vaccines (PCT/GB2012/000467) and a patent application covering this SARS-CoV-2 vaccine. TL is named as an inventor on a patent application covering this SARS-CoV-2 vaccine and was consultant to Vaccitech. PMF is a consultant to Vaccitech and has received research funding from the Brazilian Government. AJP is Chair of the UK Department of Health and Social Care's Joint Committee on Vaccination and Immunisation, but does not participate in policy advice on SARS-CoV-2 vaccines, and is a member of the WHO Strategic Advisory Group of Experts. AVSH is a cofounder of and consultant to Vaccitech and is named as an inventor on a patent covering design and use of ChAdOx1-vectored vaccines (PCT/GB2012/000467). SF is a consultant to Immunocore. GRS has received funding from Schmidt Futures and Wellcome Trust, consulting fees from GSK Vaccines Strategic Advisory Board, has patents on SARS-CoV-2 monoclonal antibodies, has leadership roles on Oxford University Council and Oxford University Hospitals NHS Foundation Trust, and holds stock in GSK. KP reports grants from the UK Medical Research Council UK Research and Innovation and National Institute of Health Research (NIHR) Vaccine Taskforce for RNA vaccine trial, COVAC1, and honoraria for Sanofi strategic advisory boards. All other authors declare no competing interests.
