# Supplementary material for: Safety and immunogenicity of the ChAdOx1 nCoV-19 (AZD1222) vaccine against SARS-CoV-2 in HIV infection: a single-arm substudy of a phase 2/3 clinical trial
Source: Lancet HIV. 2021 Jun 18;8(8):e474–85. doi: 10.1016/S2352-3018(21)00103-X (PMC8213361; doi:10.1016/S2352-3018(21)00103-X)

THE LANCET
HIV

Supplementary appendix

This appendix formed part of the original submission and has been peer reviewed. We post it as supplied by the authors.

Supplement to: Frater J, Ewer KJ, Ogbe A, et al. Safety and immunogenicity of the ChAdOx1 nCoV-19 (AZD1222) vaccine against SARS-CoV-2 in HIV infection: a single-arm substudy of a phase 2/3 clinical trial. *Lancet HIV* 2021; published online June 18. http://dx.doi.org/10.1016/S2352-3018(21)00103-X.

Supplementary Material

Appendix 1. Oxford Trial Vaccine Group

Marion E. E. Watson	Dr	Jenner Institute, Nuffield Department of Medicine, University of Oxford	PhD	marion.watson@ndm.ox.ac.uk
Rinn Song	Dr	Oxford Vaccine Group, Department of Paediatrics, University of Oxford, UK	MD	rinn.song@paediatrics.ox.ac.uk
Paola Cicconi	Dr	Jenner Institute, Nuffield Department of Medicine, University of Oxford, UK	PhD	paola.cicconi@ndm.ox.ac.uk
Angela M. Minassian	Dr	Jenner Institute, Nuffield Department of Medicine, University of Oxford, UK	PhD	angela.minassian@ndm.ox.ac.uk
Sagida Bibi	Dr	Oxford Vaccine Group, Department of Paediatrics, University of Oxford, UK	PhD	sagida.bibi@paediatrics.ox.ac.uk
Simon Kerridge	Mr	Oxford Vaccine Group, Department of Paediatrics, University of Oxford, UK	MSc	simon.kerridge@paediatrics.ox.ac.uk
Nisha Singh	Dr	Oxford Vaccine Group, Department of Paediatrics, University of Oxford, UK	DPhil	nisha.singh@paediatrics.ox.ac.uk
Catherine M. Green	Dr	Clinical BioManufacturing Facility, Jenner Institute, University of Oxford , UK	PhD	cbfhead@ndm.ox.ac.uk
Alexander D Douglas	Professor	Jenner Institute, Nuffield Department of Medicine, University of Oxford, UK	DPhil	sandy.douglas@ndm.ox.ac.uk
Alison M Lawrie	Dr	Jenner Institute, Nuffield Department of Medicine, University of Oxford	PhD	alison.lawrie@ndm.ox.ac.uk
Elizabeth A Clutterbuck	Dr	Oxford Vaccine Group, Department of Paediatrics, University of Oxford	PhD	elizabeth.clutterbuck@paediatrics.ox.ac.uk

Appendix 2 – Supplementary Tables and Figures

Table S1. Incidence of solicited adverse events in PWH and HIV-ve participants during the 7 days after prime and boost ChAdOx1 nCoV-19 vaccination

Systemic/ local	Symptom	Group	Prime		Boost	
			Any	None	Any	None
Systemic	Feverish	PWH	10/53 (18.9%)	43/53 (81.1%)	3/51 (5.9%)	48/51 (94.1%)
		HIV negative	22/50 (44.0%)	28/50 (56.0%)	5/49 (10.2%)	44/49 (89.8%)
	Fever	PWH	0/53 (0.0%)	53/53 (100.0%)	0/50 (0.0%)	50/50 (100.0%)
		HIV negative	13/50 (26.0%)	37/50 (74.0%)	0/48 (0.0%)	48/48 (100.0%)
	Chills	PWH	12/53 (22.6%)	41/53 (77.4%)	4/51 (7.8%)	47/51 (92.2%)
		HIV negative	18/50 (36.0%)	32/50 (64.0%)	7/49 (14.3%)	42/49 (85.7%)
	Joint pain	PWH	5/53 (9.4%)	48/53 (90.6%)	5/51 (9.8%)	46/51 (90.2%)
		HIV negative	17/50 (34.0%)	33/50 (66.0%)	3/49 (6.1%)	46/49 (93.9%)
	Muscle ache	PWH	19/53 (35.8%)	34/53 (64.2%)	7/51 (13.7%)	44/51 (86.3%)
		HIV negative	26/50 (52.0%)	24/50 (48.0%)	17/49 (34.7%)	32/49 (65.3%)
	Fatigue	PWH	25/53 (47.2%)	28/53 (52.8%)	15/51 (29.4%)	36/51 (70.6%)
		HIV negative	38/50 (76.0%)	12/50 (24.0%)	27/49 (55.1%)	22/49 (44.9%)
	Headache	PWH	25/53 (47.2%)	28/53 (52.8%)	12/51 (23.5%)	39/51 (76.5%)
		HIV negative	33/50 (66.0%)	17/50 (34.0%)	15/49 (30.6%)	34/49 (69.4%)
	Malaise	PWH	18/53 (34.0%)	35/53 (66.0%)	9/51 (17.6%)	42/51 (82.4%)
		HIV negative	21/50 (42.0%)	29/50 (58.0%)	14/49 (28.6%)	35/49 (71.4%)
	Nausea	PWH	4/53 (7.5%)	49/53 (92.5%)	4/51 (7.8%)	47/51 (92.2%)
		HIV negative	13/50 (26.0%)	37/50 (74.0%)	4/49 (8.2%)	45/49 (91.8%)
Local	Pain	PWH	26/53 (49.1%)	27/53 (50.9%)	10/51 (19.6%)	41/51 (80.4%)
		HIV negative	31/50 (62.0%)	19/50 (38.0%)	24/49 (49.0%)	25/49 (51.0%)
	Redness	PWH	0/53 (0.0%)	53/53 (100.0%)	0/51 (0.0%)	51/51 (100.0%)
		HIV negative	0/50 (0.0%)	50/50 (100.0%)	1/49 (2.0%)	48/49 (98.0%)
	Warmth	PWH	6/53 (11.3%)	47/53 (88.7%)	3/51 (5.9%)	48/51 (94.1%)
		HIV negative	8/50 (16.0%)	42/50 (84.0%)	6/49 (12.2%)	43/49 (87.8%)
	Itch	PWH	2/53 (3.8%)	51/53 (96.2%)	1/51 (2.0%)	50/51 (98.0%)
		HIV negative	2/50 (4.0%)	48/50 (96.0%)	6/49 (12.2%)	43/49 (87.8%)
	Swelling	PWH	1/53 (1.9%)	52/53 (98.1%)	0/51 (0.0%)	51/51 (100.0%)
		HIV negative	0/50 (0.0%)	50/50 (100.0%)	0/49 (0.0%)	49/49 (100.0%)
	Induration	PWH	1/53 (1.9%)	52/53 (98.1%)	0/51 (0.0%)	51/51 (100.0%)
		HIV negative	0/50 (0.0%)	50/50 (100.0%)	0/49 (0.0%)	49/49 (100.0%)
	Tenderness	PWH	33/53 (62.3%)	20/53 (37.7%)	22/51 (43.1%)	29/51 (56.9%)
		HIV negative	38/50 (76.0%)	12/50 (24.0%)	30/49 (61.2%)	19/49 (38.8%)

‘Any’ refers to any occurrence of the solicited symptom, regardless of reported severity (mild-severe).

Table S2. Comparison between groups of the incidence of solicited adverse events during the 7 days after prime and boost ChAdOx1 nCoV-19 vaccination

Solicited symptom	Dose	PWH	HIV-ve	p-value*
Systemic	Prime	40/53 (75.5%)	43/50 (86.0%)	0.1770
	Boost	22/51 (43.1%)	32/49 (65.3%)	0.0262
Local	Prime	40/53 (75.5%)	44/50 (88.0%)	0.1013
	Boost	24/51 (47.1%)	37/49 (75.5%)	0.0035

*p-values from Chi-squared tests

Table S3 ELISA responses in PWH and HIV negative participants

Time point	Group	n	Median [IQR]	GMT (95% CI)	GMR (95% CI)	p-value*	p-value**
0	PWH	54	7 [6, 13]	9 (7, 11)	3.59 (2.29, 5.64)	<0.0001	-
	HIV -ve	49	2 [1, 5]	2 (2, 4)	ref		
14	PWH	54	75 [27, 156]	100 (59, 171)	1.41 (0.73, 2.71)	0.9789	<0.0001
	HIV -ve	49	76 [35, 153]	71 (48, 106)	ref		
28	PWH	52	180 [87, 410]	224 (148, 338)	1.05 (0.63, 1.75)	0.8038	<0.0001
	HIV -ve	48	174 [129, 364]	214 (156, 293)	ref		
42	PWH	50	1440 [704, 2728]	1498 (1147, 1956)	2.04 (1.48, 2.81)	0.0001	<0.0001
	HIV -ve	46	736 [497, 1120]	736 (611, 887)	ref		
56	PWH	49	941 [531, 1445]	1025 (774, 1356)	1.66 (1.14, 2.41)	0.0161	<0.0001
	HIV -ve	47	631 [338, 1037]	616 (478, 795)	ref		

*p-value from Wilcoxon rank-sum/Mann Whitney test between PWH and HIV-ve groups at each timepoint

**p-value from Wilcoxon signed-rank test in the PWH group, comparing responses at each timepoint to the day 0 ELISA (tests not performed for HIV-ve group)

GMT Geometric Mean Titre; GMR Geometric Mean Ratio; CI Confidence Interval. PWH People with HIV.

Table S4 ELISpot responses in PWH and HIV negative participants

Time point	Group	n	Median [IQR]	GM (95% CI)	GMR (95% CI)	p-value*	p-value**
0	PWH	47	68 [48, 121]	90 [73,112]	14.9	0.25	-
	HIV -ve	32	52 [48,108]	80 [61.4, 104]	12.5		
14	PWH	44	674 [341, 1223]	624 [472, 826]	88.6	0.35	<0.0001
	HIV -ve	31	965 [373, 2203]	679 [401, 1148]	80.6		<0.0001
28	PWH	39	381 [244, 681]	401 [318, 506]	84.6	0.16	<0.0001
	HIV -ve	12	246 [68, 803]	249 [109, 570]	58.3		0.012
42	PWH	42	337 [188, 672]	349 [272, 448]	66.7	0.48	<0.0001
	HIV -ve	28	340 [97, 610]	279 [182, 428]	60.7		<0.0001
56	PWH	39	333 [191, 564]	305 [230, 406]	69.2	-	<0.0001
	HIV -ve	-	-	-	-		-

A positive response was defined as >221 SFCs/million PBMCs. *p-value from Wilcoxon rank-sum/Mann Whitney test between PWH and HIV-ve groups at each timepoint
**p-value from Wilcoxon signed-rank test in the PWH group, comparing responses at each timepoint to the day 0 ELISPOT.
GMT Geometric Mean Titre; CI Confidence Interval. PWH People with HIV.

Figure S1. Gating Strategy for T cell proliferation Panel

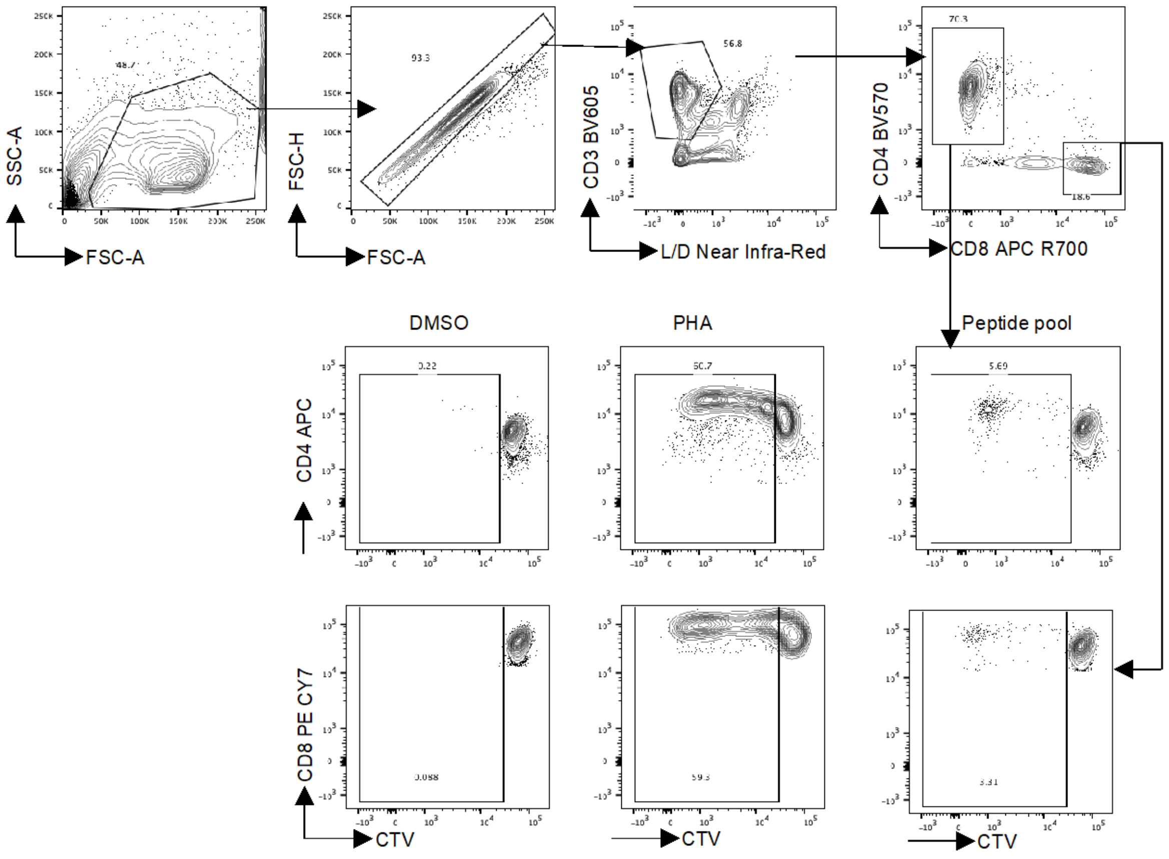

Figure S2. Gating Strategy for T cell Activation Panel

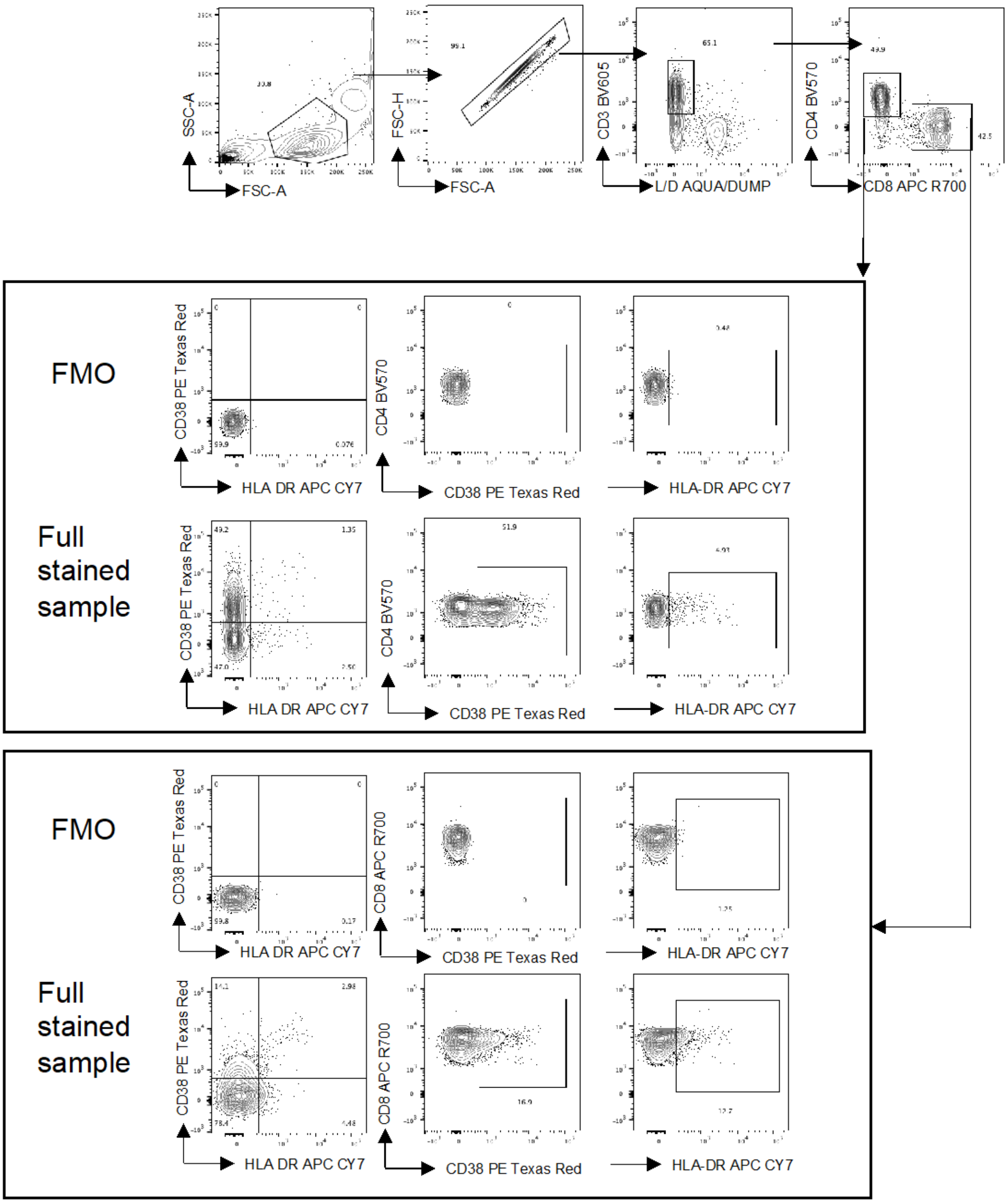

Figure S3 Correlation of ELISA with Neutralisation (Day 56 after vaccination)

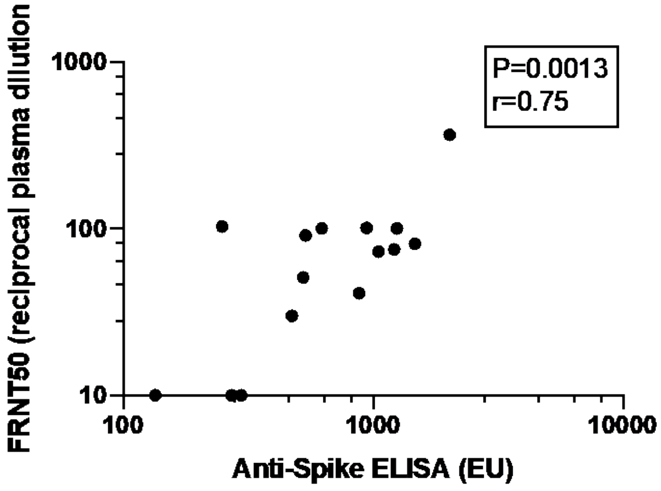

Figure S3. Anti-spike ELISA correlates with FRNT50 at day 56 after vaccination in the sub-group of 15 individuals tested for neutralisation. Pearson’s correlation.

Figure S4. Proliferative responses to FECT pooled antigens and PHA

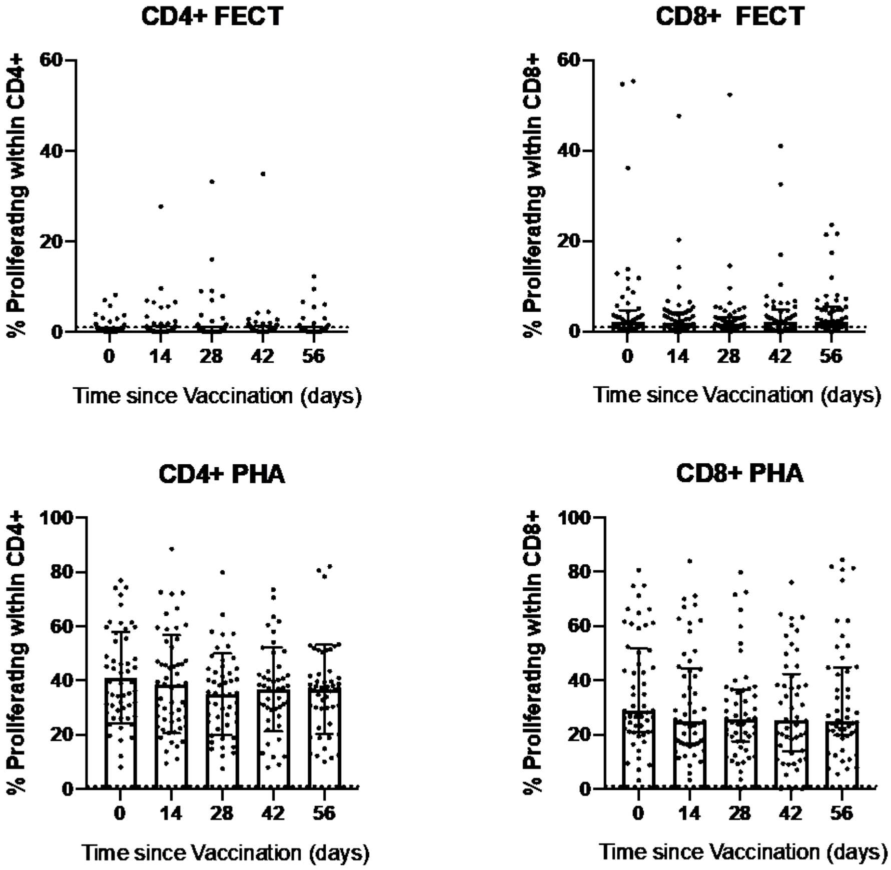

Figure S4. Proliferative responses of CD4+ and CD8+ T cells to the control FECT and PHA antigens. Responses are sustained across the 56 days for all, and there is no impact of vaccination.

Figure S5. Distribution of baseline serology vs ELISpot responses

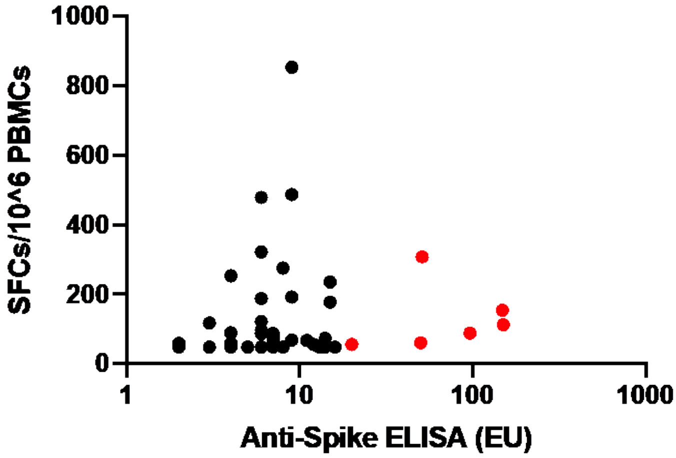

Figure S5. Distribution of baseline ELISpot and ELISA data. The six individuals with raised baseline serology are shown in red, and are not significantly associated with raised ELISpot responses.

Figure S6. Correlation matrix of impact of baseline CD4+ and CD8+ T cell activation on vaccine responses.

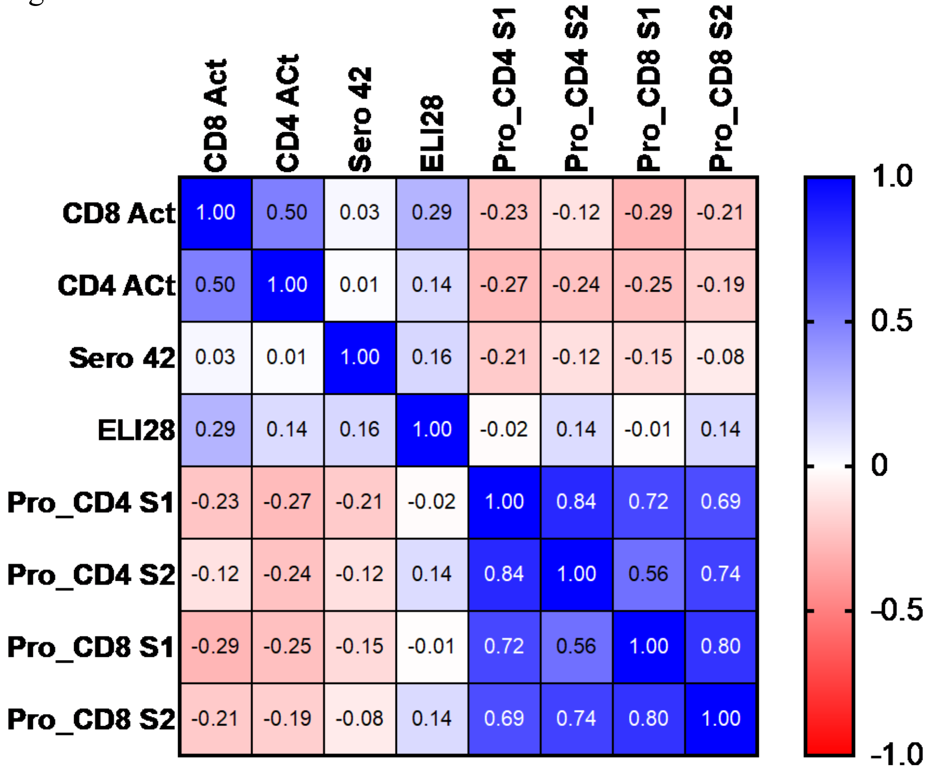

Figure S6. Pearson r values for interaction between CD8+ and CD4+ T cell activation and vaccine responses (Serology Day 42, ELISpot Day 28, Proliferation of CD4+ and CD8+ T cells to peptides pools S1 and S2 (Day 42). No interaction with activation had P value <0.05.

Figure S7 CD4 T Cell Counts amongst Participants.

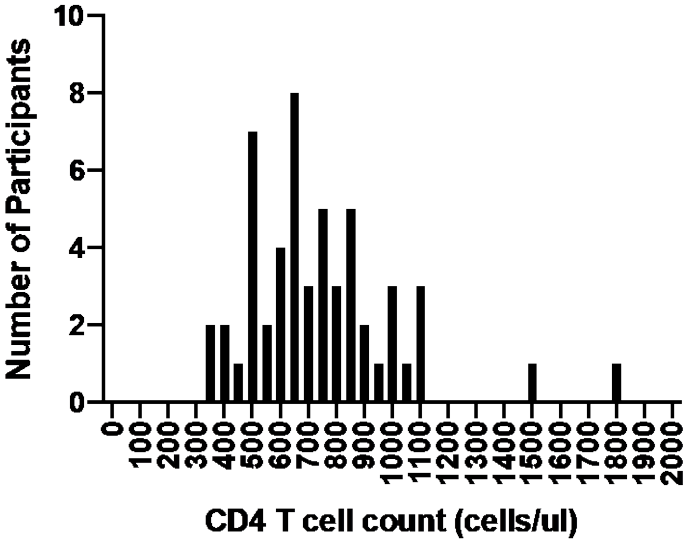

Figure S8 CD4 and CD8 T cell activation following vaccination.

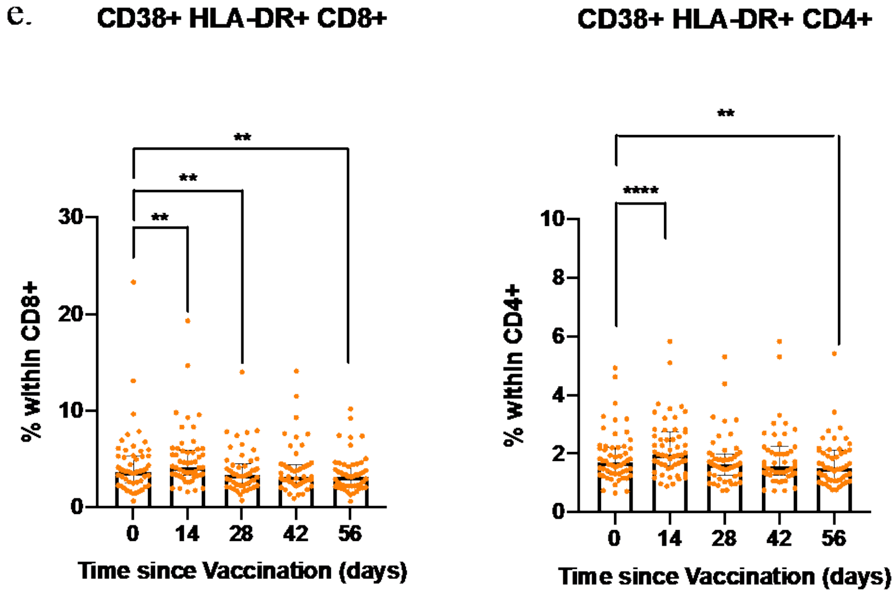

Supplement: Supplementary appendix [file mmc1.pdf]
